# Back to the Future: Very High-Energy Electrons (VHEEs) and Their Potential Application in Radiation Therapy

**DOI:** 10.3390/cancers13194942

**Published:** 2021-09-30

**Authors:** Maria Grazia Ronga, Marco Cavallone, Annalisa Patriarca, Amelia Maia Leite, Pierre Loap, Vincent Favaudon, Gilles Créhange, Ludovic De Marzi

**Affiliations:** 1 Centre de Protonthérapie d’Orsay, Department of Radiation Oncology, Campus Universitaire, Institut Curie, PSL Research University, 91898 Orsay, France; mariacrazia.ronga@curie.fr (M.G.R.); marco.cavallone@curie.fr (M.C.); annalisa.patriarca@curie.fr (A.P.); amelia.maialeite@curie.fr (A.M.L.); pierre.loap@curie.fr (P.L.); gilles.crehange@curie.fr (G.C.); 2 Thales AVS Microwave & Imaging Sub-Systems, 78141 Vélizy-Villacoublay, France; 3 INSERM LITO U1288, Campus Universitaire, Institut Curie, PSL Research University, University Paris Saclay, 91898 Orsay, France; 4 INSERM U 1021-CNRS UMR 3347, Campus Universitaire, Institut Curie, PSL Research University, University Paris Saclay, 91898 Orsay, France; vincent.favaudon@curie.fr

**Keywords:** radiation therapy, very high-energy electrons, accelerators, ultra-high dose rate FLASH therapy

## Abstract

**Simple Summary:**

The development of innovative approaches that would reduce the sensitivity of healthy tissues to irradiation while maintaining the efficacy of the treatment on the tumor is of crucial importance for the progress of the efficacy of radiotherapy. Recent methodological developments and innovations, such as scanned beams, ultra-high dose rates, and very high-energy electrons, which may be simultaneously available on new accelerators, would allow for possible radiobiological advantages of very short pulses of ultra-high dose rate (FLASH) therapy for radiation therapy to be considered. In particular, very high-energy electron (VHEE) radiotherapy, in the energy range of 100 to 250 MeV, would be particularly interesting both from a ballistic and biological point of view for the establishment of this new type of irradiation technique. In this review, we examine and summarize the current knowledge on VHEE radiotherapy and provide a synthesis of the studies that have been published on various experimental and simulation works.

**Abstract:**

The development of innovative approaches that would reduce the sensitivity of healthy tissues to irradiation while maintaining the efficacy of the treatment on the tumor is of crucial importance for the progress of the efficacy of radiotherapy. Recent methodological developments and innovations, such as scanned beams, ultra-high dose rates, and very high-energy electrons, which may be simultaneously available on new accelerators, would allow for possible radiobiological advantages of very short pulses of ultra-high dose rate (FLASH) therapy for radiation therapy to be considered. In particular, very high-energy electron (VHEE) radiotherapy, in the energy range of 100 to 250 MeV, first proposed in the 2000s, would be particularly interesting both from a ballistic and biological point of view for the establishment of this new type of irradiation technique. In this review, we examine and summarize the current knowledge on VHEE radiotherapy and provide a synthesis of the studies that have been published on various experimental and simulation works. We will also consider the potential for VHEE therapy to be translated into clinical contexts.

## 1. Introduction

Cancer is one of the leading causes of disease/death worldwide, with around 14 million new cases and 8 million deaths each year. It is forecast that the incidence of newly diagnosed cancer cases worldwide will significantly increase from today’s 18.1 million to 29.5 million by 2040 [1]. Radiotherapy has the potential to benefit approximately 50% of cancer patients during the course of their disease. However, major challenges remain as survival rates differ starkly between different cancer types—just 5% of those with lung cancer survive for 10 years, with survival from pancreatic cancer barely improving at all, regardless of the radiotherapy technique used on these cases. The development of innovative approaches that would reduce the sensitivity of healthy tissues to irradiation while maintaining the efficacy of the treatment on the tumor is therefore of crucial importance for the progress of the efficacy of radiotherapy.

The application in radiation therapy of new protocols based on the radiobiological advantages of very short pulses of ultra-high dose rate (FLASH) therapy [2] could be facilitated by recent methodological developments and innovations, such as scanned beams, ultra-high dose rates, and very high-energy electrons, which may be simultaneously available on new generations of accelerators. Besides, recent advances in terms of compactness and performance of accelerator technologies with high-gradient cavities make it possible to envisage in the short term the advent of such machines in clinical environments.

Very high-energy electron (VHEE) radiotherapy, in the energy range of 100 to 250 MeV, first proposed in the 2000s, would be particularly accurate and minimally affected by tissue heterogeneities (unlike low-energy electrons or photons), and could be applicable in a large number of deep anatomical localizations [3]. It is also potentially less expensive than particle therapy techniques, and would allow for accelerated treatment, for example through electromagnetic scanning of charged particle beams, with high doses per fraction, thereby improving its effectiveness. It is also possible to take advantage of recent work on FLASH—in which a high dose is administered to the tissues in an extremely short time—allowing for a simultaneous reduction in the occurrence and severity of early and late complications affecting normal tissues, while maintaining control of the tumor.

In this review, we examine and summarize the current knowledge on VHEE radiotherapy and provide a synthesis of the studies that have been published on various experimental and simulation works. We also consider the potential for VHEE therapy to be translated into clinical contexts.

## 2. History and Reputation of Electrons

### 2.1. Short History of Electron Radiotherapy

The history of electrons in radiotherapy dates back to the early 1950s if we consider the first treatments on medical linear accelerators. It has undergone numerous developments, in parallel with the evolution of technologies and methods of cancer treatment. For example, electron beam radiation therapy has been an important part of treatment for breast or chest wall irradiation [4], and then gradually limited itself to more specific techniques. It has also long been favored for treatments of the skin, eyes, salivary glands or part of the breasts, and often considered as a complementary method to the use of X-rays [5]. Indeed, due to their remarkable advantages over photon beams, i.e., high surface dose and rapid dose fall-off beyond maximum depth, electron beams between 5 and 20 MeV were commonly used for the treatment of superficial malignancies. Some groups have also worked on the adaptation of multi-leaf collimators (MLC) by proposing to replace field shaping cut-outs, usually mounted on electron applicators close to the patient with a photon multi-leaf collimator for electron beam collimation [6], for example, to combine electrons and photons with intensity modulation in accelerated partial breast treatment [7]. The development of electron-specific MLC with thinner leaves and a shorter distance to the patient surface has also recently been proposed [8]. Another approach that can be found in the literature is called modulated electron radiation therapy (MERT), which is based on energy and intensity modulation of the electron beam to conform the prescription dose to the distal edge of the tumor volume, while maintaining dose homogeneity within the target volume [9]. Moreover, as X-ray contamination could add some limitations to the advancement and clinical utility of those electron modalities, the clinical potential of scattering foil free-electron beams and potential application to breast treatments has been investigated [10]. New scattering foil designs allowing for non-uniformities in the dose profiles have been described for application in MERT [11]. Various attempts to improve electron conformal therapy have been proposed, such as electron arc therapy (EAT), MERT, or dynamic electron arc radiotherapy (DEAR), which consists of delivering radiation while the gantry rotation and dose rate are modulated [12]. Thus, all the elements for the implementation of complex intensity-modulated ERT in clinical routine seemed to be in place at the beginning of the 2010s (at least for superficial tumors), but the implementation did not finally take place, probably due to the advent of modern IMRT techniques such as tomotherapy or VMAT. Possible improvements in deep-seated tumor treatments were also considered with the use of intensity- and energy-modulated higher-energy electron beams (15–50 MeV) in IMRT treatments, which proved to be of little significance in selected clinical cases [13].

Today, one of the most common applications of electrons is the intraoperative technique (IOERT), consisting of the application of a dose during or after surgical removal of the tumor mass with high-energy electrons: for this technique, electron beams with energies between 4 and 12 MeV are typically produced [14], and designed for use in a common unshielded operating room environment. Clinical results of IOERT with favorable improvement in local control have been consistently reported in the last decades for pancreas [15] treatment or for the treatment of the breast, although the results are a little more controversial, particularly because of the lack of some long-term outcomes or evidence-based guidelines [16,17].

For a long time the accuracy of treatment-planning systems, initially based on the pencil-beam redefinition algorithm, was also limited for certain clinical applications because they could not properly model electron therapy (e.g., skin collimation, internal collimation, variable-thickness bolus, and arc therapy) [5]. In that regard, Monte Carlo dose algorithms have played a significant role in electron beam planning, as they have been shown to significantly improve dose calculation accuracy, for example with a more accurate handling of heterogeneities and irregular surface contours [18].

### 2.2. FLASH and Ultra-High Dose Rate Irradiation

Recently, low-energy electron machines and modified clinical linacs [19,20,21,22] were used to discover and confirm a new potential treatment method known as FLASH radiation therapy [2,23]. This irradiation technology involves the ultra-fast delivery of radiation treatment at dose rates several orders of magnitude greater than those currently used in clinical practice. It has recently been shown that FLASH with electrons (but also photons or protons) was as efficient as conventional irradiation for tumor inhibition, while dramatically less damaging to healthy tissue [2,24]. The main, characteristic advantage of the FLASH modality of irradiation is to spare normal tissues from the late complications classically observed after radiation therapy at conventional dose rate, while leaving the efficiency against grafted tumors unchanged. Normal tissue sparing by FLASH has been observed in several organs in mice including lung [2], intestine [25], skin [26,27,28], and brain [29,30,31,32], as well as cutaneo-muscular necrosis in rat tail [33], cat face, and pig skin [23], and it correlates with the down-regulation of radio-induced senescence and inflammatory processes [34,35]. The molecular mechanisms underlying such differences are the main challenge for future studies of the FLASH effect. While the mechanisms underlying the biological effects have yet to be elucidated, the FLASH effect has also been confirmed in a first human patient with promising results, supporting further studies and clinical trials [36]. The role of the beam parameters in triggering the effect also has yet to be elucidated: indeed, in addition to the mean dose rate, total dose, and total irradiation time, the pulsatile nature of irradiation (dose per pulse, pulse duration, instantaneous dose rate, and pulse repetition rate) may also influence the FLASH effect. In order to induce a FLASH effect, it seems that the irradiation beam should ideally be pulsed with a minimum number of pulses, allowing for a sufficiently high dose per pulse and dose rate within the pulse (e.g., >1 Gy and 10^6^ Gy/s, respectively), and implying a total irradiation time lower than 100 ms [37,38]. The pulse repetition rate should be adapted to allow for a fast scan of the target if necessary (at first glance at least a few tens of Hz), while keeping the dose per pulse high and the total time short.

However, few devices are available today to deliver FLASH radiation, and pre-clinical investigations are conducted by many research teams on machines specifically dedicated to small animals or designed for conventional clinical treatments. Tuned clinical accelerators have been used by investigators [19,39]. A whole field of research emerged with the aim of fulfilling the conditions triggering the FLASH effect with various approaches, such as the PHASER program [40] and laser-driven accelerators, which are considered to be the next generation of cost-effective accelerators for radiotherapy [41], as well as very high-energy electrons (VHEEs) exceeding 100 MeV [42] or intraoperative radiation therapy [43]. Many reviews already exist on the progress of work related to FLASH; some focus on biological aspects [44], and others are more comprehensive [45] or address translational aspects [37]. Although photons (X-rays or γ rays), protons, or electrons can be used to generate the FLASH effect, the majority of studies have been performed using electrons from linacs. As long as their energy is sufficient to ensure good penetration of the tissues, electrons could indeed have a certain number of advantages compared to other types of radiation.

## 3. Very High-Energy Electrons and Their Potential Application in Radiation Therapy

### 3.1. Many Advantages Related to the Physical and Dosimetric Properties

**Figure 1:**
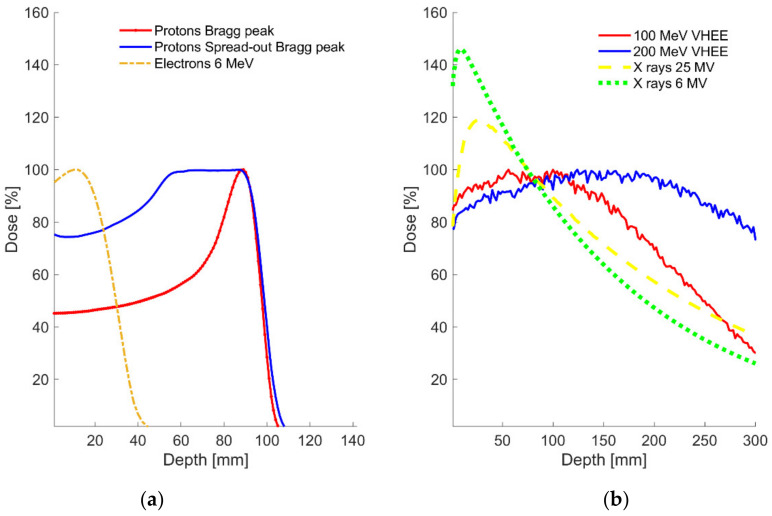
Comparison of relative depth dose distributions of photons, protons, and electrons, and VHEEs in case of a single field: (**a**) 110 MeV protons Bragg peak or SOBP and 6 MeV electrons in water, (**b**) 25 and 6 MV X-rays, 100 and 200 MeV VHEE in water (Monte Carlo simulation).

The use of VHEEs between 50 and 250 MeV for radiotherapy was proposed and studied in detail in the early 2000s (see Figure 1 for a comparison of depth dose distributions between various beams). A first series of papers demonstrated the interest of these beams from the point of view of their ballistic properties [3,46,47]. First of all, it was shown using Monte Carlo methods that electrons in the range of energies between 150 and 250 MeV were sufficient to reach the deepest tumors in a patient. Depending on the beam arrangements (single, parallel opposed, or orthogonal), the penetration of VHEE beams seemed adequate for the most deep-seated tumors, even if the penetration is facilitated when the field size increases. Indeed, there is a strong dependence of depth of the maximum dose (d_max_) on the geometrical size of the beam. The scattering of monoenergetic electron beams in air was simulated and shown to be rather moderate: the spread in air of a 0.86 mm FWHM electron beam (with 0.43 mrad angular spread) was for example increased from 2 to 7 mm between 100 and 250 MeV, respectively, after 1 m of air crossed. Indeed, the scattering power of charged particles is inversely proportional to the energy squared, which means that the lateral diffusion has a weak dependence on the energy. However, the lateral penumbra of VHEE beams deteriorates more quickly in depth and is more pronounced for larger depths and lower beam energies compared to the penumbra of MV photon beams. A short air distance (<70 cm) between the vacuum window and the patient would therefore be necessary to keep an acceptable pencil beam size when the scanned beam technique is used. Moreover, the VHEE beams proved to be relatively insensitive to surface obliquities, and the dose at the surface (or tumor-to-normal tissue dose ratio) to be less than the maximum depth dose, thus ensuring that sufficient skin tissue is spared (provided that at least two beams are used). Additionally, the authors also studied the effects of tissue heterogeneities on VHEE beams and compared them with MV photon beams using simulations. It was found that uniform dose distributions were maintained at interfaces between organs and tissues of different densities (lung, air cavities, bone, muscle, and fat) for VHEEs. These results were later confirmed by experiments conducted at the VESPER test stand of the CLEAR facility (CERN, Switzerland), with a beam of 156 MeV and 1.2 mm standard deviation. The authors showed that the longitudinal dose profiles in water of the VHEE beam were relatively unaffected (less than 5–8% dose variation) when crossing different heterogeneities with densities 0.001–2.2 g/cm^3^ [48].

Later, in the 2000s, several laboratories achieved the ability to increase the energy of laser-accelerated electron beams to more than 100 MeV and to produce quasi-monoenergetic beams [49]. New studies based on realistic beam properties for this type of accelerator were then conducted (beam energy between 150 and 250 MeV, 15 MeV energy spread, and 6 mrad FWHM initial angular distribution), showing that the dosimetric properties of the laser-accelerated VHEE beams would also be suitable for the treatment of deep-seated tumors, and that magnetic focusing of the electron beam would improve the lateral penumbra [50]. The possibility of converging 160 MeV VHEE beams in water, decreasing the beam width from 7.7 to 3.0 mm on one axis, was then demonstrated experimentally at the CLEAR facility using external magnetic fields and two electromagnetic quadrupole triplets [51]. This approach has also been strengthened by MC simulations, and experimentally demonstrated at 158 and 201 MeV by another group [42,52], allowing for laterally focused beams to be obtained (FWHM between 2 and 6 mm). The depth dose curves of these symmetrically focused electrons also show a depth peak and a more favorable dose distribution with reduced proximal and distal doses. Finally, the possibility to shape both the on-axis and transverse plane dose distributions of the beam, as well as to create weighted sums of focused electron beams to form spread-out electron peaks (SOEP) over a target region from focused beams, was demonstrated in a very recent study [53].

Other teams involved in the development of spatially fractionated radiotherapy (SFRT) have proposed to exploit the lateral scattering properties of very high-energy electrons between 150 and 300 MeV (at least with focused beams) to generate minibeams. Indeed, spatial fractionation has the potential to considerably reduce radiation toxicity, while achieving tumor control equivalent or superior to that of conventional radiation therapy. In particular, GRID therapy has been employed successfully in the palliative treatments of large and bulky tumors with early phase clinical trials showing remarkable success [54], and the minibeam (which uses sub-millimetric field sizes—400–700 µm) showed a remarkable increase in sparing of normal tissue in preclinical studies [55]. Thus, it was shown by Monte Carlo simulations that very large values of PVDR (>2000) could be obtained with focused VHEEs of at least 3 mm width (in order to keep a sufficient penetration of electrons and depth of the maximum dose) and 3 mrad divergence [56]. In the 1980s, the idea of combining high dose rate, high-energy particles, and spatial fractionation or GRID therapy to better preserve healthy tissue had already emerged: “the low skin reaction from high pulse and dose rate reported by Griem et al. may be combined with a favourable GRID technique” [57].

Lastly, in another recent paper [58], magnitudes and dependencies of VHEE beam ranges and penumbra as a function of energy, field size, and source-axis distance (SAD) were simulated for a broad beam (from a point source with various source-to-surface distances). This work showed for example the complex relationships between divergence and beam energy and various clinical quantities (such as the skin dose, the depth of the maximum dose, the extent of the dose region above 90%, or the field size), pointing out the importance of having high-energy and large SADs in order to achieve optimal penumbra and PDD for the treatment of deep clinical targets.

### 3.2. Conformation Techniques

There are two established methods by which the electron fluence of the beam can be spread over a larger area. One is called multiple scattering (usually in one or two foils made of a high-Z material), which allows for the production of a broad beam to match the maximum lateral tumor dimension. The other one is called active scanning or pencil beam scanning (PBS), and consists of the electromagnetic scanning (in steps or by continuous shift) of several small pencil beams over the target volume [59]. The development of scanned VHEE beams does not seem extremely complicated since the technique has existed for decades with electrons and has even become very mature for high-energy clinical protons (100–250 MeV). However, the modality that would truly push VHEEs into clinical deployment would be their delivery in the FLASH irradiation condition. The main difficulty of this mode of irradiation is that the specification on the minimum dose rate requires the prescribed dose to be delivered to a 1 L volume (e.g., 10 × 10 × 10 cm^3^) in 100 ms for a single field, while having a pulsed beam delivery output. For 1 Gy, this equates to 1.35 × 10^11^ electrons at 100 MeV, equivalent to 21.6 nC of charge or 216 nA average current. If the beam were to be pulsed with a 1 µs pulse duration, this would require a peak current of 21.6 mA for 1 Gy in 1 µs pulse.

The simplest way to meet the above requirement would be the traditional scattering system for which there are certain limitations: (i) a collimation system such as multi-leaf collimator (MLC) would be needed to provide an alternative to the manufacturing of patient-specific collimators, but no clinical systems currently exist at very high energy; (ii) there will be a significant increase in photon/neutron dose to the patient as a result of the extra beam losses in the scatterer/collimators/beam diagnostics; (iii) transmission losses will increase with the field size and no efficient scattering system exists for VHEE beams. Some design aspects of VHEE beam shaping were also discussed by [60], such as the improved ballistic properties (penumbra, coverage of the tumor) in case of a non-divergent beam, or the importance of a low-energy spread when using magnetic fields to make the beam parallel. Besides, the current clinical foils, which were designed to produce flat beams for low-energy electron beams, are not designed for VHEE applications, and even less so for FLASH. In the context of the optimization of their scattering system for FLASH ERT (<6 MeV), some authors have proposed to use stainless steel and aluminum for the primary and secondary foil materials, respectively, and optimized their thicknesses (0.075 and 0.3 mm, respectively) in order to keep a ultra-high dose rate over a 5 × 5 cm^2^ field for a given SAD [61]. This type of empirical approach or the more theoretical one of [62], which presented a detailed theoretical model to deduce the minimum total scatterer thickness as well as shape of the second scatterer for any given particle type, energy, and field size, and could allow for the determination of the feasibility and limitations of very high-energy scattering systems.

For comparison, as there is a factor of about 3–4 between the magnetic rigidity of protons and electrons of similar energy [47], a scanning system for VHEEs could be more compact than for protons (with magnetic fields <1 T, <2 m of space for the scanning magnets, and length to get lateral displacement of the beam). As the time to move the beam is proportional to the inductance in the scanning magnets, the magnetic field to current parameter, and the magnetic rigidity, the compactness of the scanning system as well as the magnets’ parameters can thus be important to respect the specifications on the dose rate in FLASH mode. In a scanning system with a step and shoot approach, and for the aforementioned 1L volume irradiated in less than 100 ms, each field would contain 2500 pencil beams (assuming a 2 mm spacing between spots): a scanning speed of at least 5.1 m/s would then be necessary (assuming no pause related to magnet stabilization or dose control during irradiation). However, this would imply a minimum repetition rate of 25 kHz, which does not exist for linacs. FLASH-VHEE radiotherapy with a scanned beam can therefore only exist if small sub-volumes (whose sizes remain to be determined) of irradiated healthy tissues can be treated at FLASH rates without the entire volume needing to be treated within 100 ms: this is also the current concern for FLASH proton beams [63].

### 3.3. Biological Specificities of High-Energy Electrons

In the 1960s, and with the pioneering use of the first electron beams (in particular at the University of Chicago), several cohorts of patients were treated with scanned electron beams between 3 MeV and 50 MeV. Several observations were reported that suggested that the skin reactions were less than those reported in conventional conditions [64,65]. The authors analyzed the temporal structure of the beam, noting that in 0.45 s, a 20 µm cell would receive 27 pulses of 1 µs duration with an instantaneous dose rate of 10^6^ Gy/s, much more than with scattered beams, and that these high dose rates may play a part in the very limited skin reactions [66]. 

At the present time, experimental in vivo and clinical data have shown that a generic RBE of unity for the value of low LET/energy photons and electrons seems to be appropriate, although there are many factors that influence RBE values including characteristic spectral LET distribution of the reference radiation, filtration, cell type, and the biological endpoint under consideration [67]. Indeed, on the one hand, there is some recent experimental evidence that large doses and dose rate variations may play a significant role in determining the response of some organs and tissues to irradiation (see Section 2.1 about the FLASH effect). On the other hand, major differences have recently been observed between scattered or scanned beams (in particular for protons), such as in late skin gene expression or according to the organs considered in terms of genotoxicity, antioxidant capacity, or inflammatory cytokines [68,69], which could also be related to the dose rate even if we are not in the application range of the FLASH effect. The role of the immune system and the microenvironment in the response to radiation exposure is also mentioned as a possible explanation of the FLASH effect [44]. The impact of direct killing of circulating immune cells, dependent on the fractions of the cardiac output and blood volume exposed, would therefore also be very dependent on the type of irradiation (passive or scanned) and dose rate or temporal fractionation of the dose.

Although many studies exist on the relative biological effectiveness (RBE) of electrons up to 35–50 MeV, very few data exist for higher energies and even fewer for VHEE beams. In the study conducted by [70], the theoretical RBE for VHEE beams between 100 and 300 MeV was evaluated using the lineal energy spectra obtained from MC simulations as inputs to the microdosimetric kinetic model (MKM). No difference in the calculated RBE was found compared to conventional 20 MeV electron beams, although the dose-averaged linear energy transfers (LET_d_) for VHEEs were estimated to be slightly higher (0.4–0.8 keV/µm) than those of the low-energy electrons (0.255 keV/µm). In another recent study [71], the first plasmid irradiation experiments were performed using VHEEs between 100 and 200 MeV at the CLEAR facility, showing little variation in double strand-break (DSB) induction with beam energy, and no statistically different variation in DSB yield between conventional or ultra-high dose rates. In that study, Geant4-DNA MC simulations were also carried out and showed that theoretical RBE (evaluated at 0.22 keV/µm for VHEE beams), physical damage to DNA, and RBE_DSB_ calculations were similar between VHEEs, conventional ^60^Co X-rays, and low-energy electrons.

Clinical electron beams also contain an admixture of the bremsstrahlung photon component produced in the treatment head (which is usually dominant for accelerators equipped with scattering foils) and in the patient. This component can increase from 0.5% to 8% of the absorbed dose (at the maximum depth dose) between 10 and 50 MeV, but of course depends a great deal on the design of the machines [72]. In order to verify if the photonuclear reactions of photons (whose maximum cross-sections in human tissues are above 20 MeV) could increase the RBE of very high-energy particle beams, the RBE values for high-energy scanned bremsstrahlung beams (50 MV) were estimated by microdosimetric and radiobiological measurements, and values up to 1.1–1.2 were found [73,74]. For VHEE beams, the absorbed dose component due to photoneutrons and charged particles from photonuclear reactions (depending on the proportion of the photon spectrum above the photonuclear reaction thresholds) could therefore be non-negligible and have an impact on the biological dose.

### 3.4. Treatment Planning Comparisons

Several studies have compared possible VHEE treatment plans to currently used techniques, such as volumetric-modulated arc therapy (VMAT) or intensity-modulated radiation therapy (IMRT) for photons, and pencil beam scanning (PBS) for protons for various clinical cases.

When compared to IMRT (15 MV), scanned VHEE (200 MeV) treatments plans have shown reduced integral doses and organ at risk (OAR) doses (by around 10%) as well as better conformity for a prostate case [46]. These results were confirmed by a second, more comprehensive prostate study, which showed that the scanned VHEE plans resulted in better dose sparing of both the rectum and bladder, as well as resulting in a lower integral dose to the normal tissues. The authors also found that an electron energy greater than 100 MeV was preferable for that case, and a large number of beams in the range of 9–21 from the fixed gantry angle position was needed to achieve acceptable plans, which were not significantly improved using arc therapy or energy modulation [75]. A second study comparing 15 MV IMRT, 250 MeV VHEEs, and a two-beam IMPT plan with 200 MeV protons showed that, for the same prostate case, intensity-modulated protons always spared more healthy tissue (between 15% and 20% of the prescribed target dose) but had a conformation relatively comparable to VHEEs. VHEEs, on the other hand, allowed for a slightly higher mean target dose, a greater target dose homogeneity, and significantly greater dose sparing of the sensitive structures compared to photons [76]. Similar results were found by another study [50], which used a clinically approved seven-field prostate treatment plan with 6 MV photons and VHEEs between 150 and 250 MeV. A 15 MV VMAT plan was also compared to a 100 MeV scanned VHEE plan, showing a VHEE dose distribution for prostate case similar to the clinical VMAT plan [77]. The VHEE plan becomes significantly better than for VMAT when the electron energy is increased to 200 MeV [78].

For a pediatric intracranial case, the 100 MeV scanned VHEE dose to all critical organs was up to 70% lower than the clinical 6 MV VMAT dose for the same target coverage, and the integral dose was also decreased by 33% compared to the VMAT plan. The optimization of the VHEE plans proved sufficient when 13 beams and more than 100 MeV were used [77]. A 100 MeV VHEE lung plan was also compared to a 6 MV VMAT plan, resulting in mean dose decrease to all OARs by up to 27% for the VHEE plan. This study of various treatment plans was extended to several other clinical cases such as acoustic neuroma, liver, lung, esophagus, and anal cancer cases, with target sizes ranging from 1 cm^3^ to hundreds of cm^3^ in [79]. The cases with bigger targets benefited most from the reduction of the dose to normal tissues, while, for smaller and shallower targets, the normal tissue sparing was similar to the VMAT plans. In this study, the mean doses to OARs were on average 22% lower for the VHEE plans compared to the VMAT plans. Dose conformity was equal or superior compared to the VMAT plans and the integral dose to the body was on average 14% lower for the VHEE plans.

In the end, the VHEE plans with scanned beams are intermediate between photon VMAT and proton PBS plans for OAR sparing, except that the OAR sparing could be made comparable to protons plans for a shallower target [78]. The sparing generally increases with VHEE energy, as well as the dose conformity and homogeneity, and requires a significant number of entry points, which must be carefully optimized according to the position of the organs at risk.

### 3.5. Radioprotection Aspects

Radiation fields of concern of very high-energy electron accelerators consist mainly of photons and giant-resonance neutrons. Indeed, prompt photon fields are produced by bremsstrahlung, which are very forward peaked (increasingly so when the energy increases). Bremsstrahlung is also the dominant electromagnetic process for high-energy electron beams interacting with matter, and it increases with the energy of the beam, so this X-ray component will be larger than 10% for energies above 50 MeV. High-energy electron bremsstrahlung theories have been documented in detail for a certain range of electron energies, showing good agreement with experimental data, although small underestimations outside the 90–120 MeV range have been reported [80]. Therefore, the bremsstrahlung component generated in the treatment head or in the tissues is an important consideration from a radiation protection standpoint and for the calculation of the energy deposition in different phantom materials, which will need to be validated in the energy range of VHEEs.

Then, three main photoneutron production processes by the high-energy bremsstrahlung photons are possible (note that the neutron production is also determined by electrodivision by electrons): giant-resonance neutron production (10 MeV < E < 30 MeV), quasi-deuteron production and decay (50 MeV < E < 300 MeV), and intranuclear cascade and evaporation/photopion production (above the threshold of 140 MeV) [81]. A rapid rise in the amount of neutron production is therefore expected for electron energies in the range 10–20 MeV, followed by a slower rise above 30 MeV as the giant-resonance is the dominant process for VHEE beams. Radioactivity may also be induced in components (e.g., carbon, oxygen, and nitrogen in human tissues, in the air, or in the treatment head) that are irradiated by an electron or bremsstrahlung beam, in particular above about 10 MeV. Thus, the development of VHEEs probably requires an accurate estimation of neutron generation yields and induced radioactivity from the point of view of patient and staff radiation protection.

The yield of neutrons for electron beams was quantified analytically in the early work on VHEEs [3,47] and estimated to be around 0.03 neutron per incident electron at 150 MeV. The increased neutron dose was estimated to be around 0.2%, which corresponds to an equivalent neutron dose of 2%, taking into account a neutron quality factor of 10 in order to be conservative from a radiation protection standpoint. The increase in dose from induced radioactivity was calculated to be approximately 0.01% of the primary electron dose. The dose due to neutrons and induced radioactivity was then found to be lower than that for energies up to 50 MeV, since the predominant source of the neutron dose occurs at the giant-resonance region (<30 MeV).

Based on MC simulations, the total body neutron dose due to VHEE irradiation with pencil beam scanning was estimated to be around 1–2 orders of magnitude smaller than that for scanned proton beams and 15–18 MV photon IMRT [82]. A neutron yield of the order of 10^−5^ neutrons per incident electron was simulated by [83] for 165 MeV scanned electron beams in water, and was still an order of magnitude lower than for 20 MV nominal X-ray energy irradiation, and much lower than the previous estimates; the authors found that induced activity due to radionuclide production had a negligible effect on total dose deposition. These first estimations may, however, vary considerably depending on the type of beam delivery chosen in the future machines (type of collimation, scanned, or scattered mode), as realistic conditions are lacking for shielding calculations.

## 4. Accelerators for VHEEs

### 4.1. General Specifications

To date, most medical accelerators are based on 3 GHz, S-band cavities with accelerating gradients far below 100 MV/m. The radiation beam from these accelerators then consists of short intense pulses of a few microseconds duration at repetition rates of about 100 pulses per second. Today, thanks to the development of new technologies available at several test facilities worldwide, particularly in the framework of RF-devices for linear colliders [84], compact accelerating structures with more than 100 MV/m gradients could make VHEEs a real option for cancer treatment. The progress made to achieve high accelerating gradients with X-band RF structures at 12 GHz could even be enhanced using plasma Wakefield-based acceleration, as we discuss in the following sections [85]. Indeed, to perform VHEE radiation therapy, an electron source should be very compact, reliable, and able to cover large irradiation areas (transverse field sizes > 10 × 10 cm^2^). In addition, VHEEs would have to be delivered rapidly and with a very intense beam (high luminosity is also needed for colliders), in a very controlled and robust way. In this regard, several test facilities worldwide are currently identified as experimental platforms able to provide the physical and the pre-clinical environment for such innovative RT modalities. From the point of view of depth dose distributions, an accuracy of ±10 MeV may be quite reasonable for a VHEE accelerator, as small fluctuations in beam energy have a minimal effect on the absorbed depth dose. However, if the type of machine requires the beam to be focused or deflected by magnetic fields, the energy spread should be kept as small as possible (<1%) in order to maintain clinically acceptable transverse parameters (e.g., spot size), which are specifications that can only be met in a small number of facilities.

### 4.2. Linacs

Betatrons or racetrack microtrons were first considered as potentially capable of delivering VHEE [47], but it was the linacs that became established in clinical routine and then as capable of being upgraded to deliver very high energy. There are three main facilities that can be used today (described briefly below, whose main parameters are reported in Table 1), and several more are planned.

Located at CERN (Switzerland), the probe-beamline of the CLIC Test Facility was converted in 2017 into the CERN Linear Electron Accelerator for Research (CLEAR) [86]. This 25-meter-long linear accelerator produces bunched electron beams from a photocathode coated with cesium telluride, and after three S-band acceleration structures, the beam achieves energy of about 220 MeV. Two irradiation areas are available for users to study X-band RF components (typically around 12 GHz) and novel concepts such as the use of plasma or THz-wavelength radiation for charged-particle acceleration, but also the radiation hardness resistance of electronic devices and medical applications. Several studies on the use of VHEE beams for clinical employment were already conducted at CLEAR, especially in the field of dosimetry in very high dose rates conditions [51,52,71,87,88].

The Next Linear Collider Test Accelerator (NLCTA) at SLAC (USA) produces high-brightness electron beams by an S-band RF photoinjector, achieving a final energy of 120 MeV after two high-gradient X-band RF linear accelerating structures (25 m long) [89]. Three experimental areas are available for users, and in particular, experimental irradiation on VHEE dosimetry has been conducted with energy up to 70 MeV [90].

The Sources for Plasma Accelerators and Radiation Compton with Lasers and Beams (SPARC) linac at the INFN-LNF (Italy) test bench consists of a photoinjector with a Cu photocathode and three S-band travelling wave accelerating sections, achieving an energy of approximately 170 MeV [91]. To tune the beam properties in the irradiation area at the target position, eight electromagnetic quadrupoles can be used. This beamline was already employed for dosimetry measurement of VHEEs [83].

Many other facilities are developing access to VHEE beams (see Table 2 for a non-exhaustive list of facilities) to support the increasing interest of the community towards medical applications of VHEE.

The ultrabright electron beam test facility of the compact linear accelerator for research and applications (CLARA) at Daresbury (UK), built to test advanced free-electron laser (FEL) schemes, is able to deliver electron beams up to 50 MeV, with a bunch charge of 250 pC at a 10 Hz repetition rate, which was already used for pre-clinical tests [92]. The S-band photoinjector and a three-stage linear S-band accelerating structure will be upgraded in a second phase to upgrade the acceleration to energy of 250 MeV and will include a separate beamline for users.

The Photo Injector Test facility at DESY in Zeuthen (PITZ, Germany) was built to test and optimize high-brightness electron sources for FEL user facilities. At present, a maximum energy of 20 MeV can be used with ultra-high dose irradiation conditions, as it can work with a dose rate per bunch of around 4.10^13^ Gy/s. A possible upgrade to 250 MeV would make PITZ a good candidate to perform VHEE in the FLASH-RT mode [93].

Recently, the Argonne Wakefield Accelerator (AWA) test stand at Argonne National Laboratory with an energy up to 60 MeV, pulse charge of around 100 nC, and repetition frequency of 10 Hz, opened up to collaboration opportunities [94], as did the Inverse Compton Scattering Source of Tsinghua University, (Beijing, China) [95], which is under construction and plans to provide a beam energy up to 350 MeV with a bunch charge of 200 pC and a bunch length of <2 ps (see Table 2).

At present, the only accelerator solution that is being designed to produce a clinical compact system to perform image-guided FLASH-RT with photons or very high-energy electrons between 100 and 200 MeV is the PHASER system [40,82]. For this project, an innovative power-efficient linear accelerator structure and RF power sources were engineered. The distributed RF-coupling architecture with genetically optimized cell design (DRAGON) structure can provide high accelerating gradients (>100 MV/m). Sixteen fast-switching (300 ns) stationary beamlines will then provide non-coplanar highly conformal radiotherapy. Electronically scanned, highly intensity-modulated beam delivery will also be implemented using a spatially patterned electron source that will be obtained by projecting an optical image onto a photocathode. The electron ‘‘image” will then be accelerated through a high-gradient DRAGON linac, steered, and magnified to the treatment volume, thus producing an intensity-modulated treatment field.

### 4.3. Laser-Driven VHEE

Laser-plasma accelerators (LPA) can produce VHEEs through the interaction of a high-power laser pulse (10^18^ W/cm^2^) with a gaseous target. In this process, known as Laser WakeField Acceleration (LWFA), the laser pulse ionizes the gas at its leading edge and creates a plasma in which a strong travelling electrostatic gradient (100 GV/m) is formed. By properly trapping plasma electrons in the accelerating region of the travelling electric field, they can be accelerated up to the energy required for radiotherapy applications, i.e., above 50–100 MeV, in a very short accelerating region of a few millimeters. This feature has drawn attention to laser-plasma accelerators (LPA) as a possible candidate to generate VHEEs for future applications, since the extremely short accelerating distance would result in lower costs as well as more compact radioprotection structures compared to RF accelerators. Numerous LWFA mechanisms differing in the way electrons are trapped in the accelerating region of the travelling electric field have been developed in recent years [96,97,98,99,100]. Among them, ionization injection [101,102,103] is an efficient and widely used method to produce energetic electrons. A comprehensive review of the main LWFA techniques can be found in [104].

One of the attractive advantages of LPA facilities is the possibility of readily tuning the electron beam properties, such as the energy and charge per bunch, by modifying the gaseous target parameters, such as its composition and density, and/or the laser parameters [96,105]. Furthermore, recent findings on the biological effect of FLASH [2,23] and ultra-high peak dose rate irradiations [41,106] have attracted further attention in LWFA accelerators, since they could also represent a unique tool to investigate the effect of ultra-high peak dose rate on living matter. In fact, LWFA electron bunches feature a pulse duration that is an order of magnitude shorter than that of conventional RF accelerators (pico-femtoseconds vs. microseconds), which results in a much higher peak dose rate in the pulse than that reached by prototype linacs used for FLASH experiments (10^11^ vs. 10^7^ Gy/s). On the other hand, the low repetition rate of LPA limits the average electron flux and therefore the maximum achievable mean dose rate and/or the field size. Most of the experiments reported in literature were performed with commercially available 10 Hz high-power lasers and reported mean dose rates in the order of Gy/min [103,107,108,109], comparable to those employed in clinical practice over a surface of a few mm^2^ to cm^2^. The development of high repetition rate (>100 Hz) laser systems delivering an energy per pulse comparable to that of currently available 10 Hz lasers will enable the generation of high repetition rate laser-driven VHEEs [110] and an increase in the mean dose rate of one or two orders of magnitude in coming years. Moreover, achieving a higher repetition rate would also benefit the electron beam stability by averaging the shot-to-shot fluctuations, as has been demonstrated with kHz, low-energy laser-driven electrons [111]. One of the main limitations of laser-driven VHEEs, which hinders their medical application, is indeed the shot-to-shot fluctuation of the electron bunch parameters such as energy, charge, and beam pointing (in the order of the beam divergence) [112].

Current efforts are focused on enabling the transition of LPA facilities from a platform dedicated to fundamental physics research to actual machines for preclinical medical applications. To this end, recent studies aimed to find solutions to improve the stability of the beam parameters at the irradiation site and to demonstrate the feasibility of using laser-driven VHEE for radiotherapy applications by mimicking irradiation schemes that are currently employed in clinical practice [113,114] or by designing adapted gantry [113,115], which demonstrated the feasibility of using intensity modulation and multi-field irradiation schemes with laser-driven VHEEs. Reference [114] reproduced a stereotactic radiotherapy irradiation scheme with 36 entrance angles and 90 MeV focused electron beams in a cylindrical phantom of 80 mm diameter. The use of a transport system allowed for the tuning of the electron spectrum and reduction of the shot-to-shot charge fluctuations to less than 1% (compared to 5.16% fluctuation without a focusing system). They achieved a conformal dose in a bean-shaped target of 0.07 cm^3^ inside the phantom while keeping the maximum dose in a nearby circular organ at risk of 0.33 cm^3^ below 12% of the target dose. All together, these studies demonstrate that laser-driven VHEEs, for which radiobiological experiments are still lacking, are ready for preclinical studies, although further efforts on both the optimization of the laser-plasma interaction mechanisms and the beam transport techniques are still needed to reduce shot-to-shot fluctuations and improve the long-term stability of such systems.

## 5. Conclusions

As already highlighted a few years ago, VHEE beams have potential advantages for radiation therapy compared to conventional electrons or X-ray IMRT [116]. The main advantages are ballistic, related to their improved dosimetric properties in the tissues. We can mention in particular the advantages of the absence of electronic disequilibrium at interfaces and reduced sensitivity to heterogeneities. VHEEs are potentially superior to photon beams (penumbra, integral dose, ballistic), as VHEEs can be scanned at high speed, allowing for simultaneous tracking and IMRT treatment, and neutron production is relatively low and therefore not an obstacle. However, compared to the most advanced techniques, such as intensity-modulated proton therapy or VMAT, these advantages are relatively minor and may not by themselves justify the transition to a VHEE type of technology. Moreover, VHEEs could have some disadvantages, such as the fact that they do not stop in the patient and do not significantly spare healthy tissues behind the tumor; Monte Carlo simulations on the contribution of secondary particles have shown that the estimated neutron dose is lower than that for scanning beam proton therapy and IMRT, but the bremsstrahlung contribution with maximum photon energies, up to several hundreds of MeV, could also create significant shielding problems; the interplay between the scanning beam and the motion of tumors or organs can be problematic. Lastly, ultra-high dose rates would pose serious challenges regarding dosimetry and monitoring of the irradiation [116]. If we re-examine the various arguments of this discussion in the light of use in ultra-high dose rate FLASH irradiation mode, we realize that many of the above-mentioned disadvantages do not apply. Indeed, the better preservation of healthy organs, the dose rate and its interest in the presence of mobile organs are all advantages of FLASH. However, the simulations of treatment plans carried out so far are very theoretical and do not take into account the constraints of FLASH irradiation (total irradiation time < 100 ms, for example), and the presumed ballistic advantages of VHEEs will probably be tempered when it is possible to simulate realistic biologically optimized cases. Many questions also remain as to whether the FLASH effect will be maintained with VHEEs: for example, what will be the biological effect with mixed radiation of different qualities (photons + electrons), dose rates, dose per fraction, and exposure volumes, which will vary greatly spatially in the context of very high-energy, scanned pencil beams or through beams.

The infrastructures we mentioned in this document for VHEE studies rely mainly on established S-band RF technology. One of their limitations is that they consist of quite large installations (20 m for a linac + 15 m for the beam transport line), which are not very compatible with the requirement for a compact solution dedicated to radiation therapy. Indeed, one of the challenges for the manufacturers is to make systems that can fit within standard vaults (or at least not much larger) and can be installed through standard doors and mazes. This would enable most clinics to integrate such new technology into their treatment options. To meet such specifications, the use of X-band linacs is foreseen in several collaborations, as this technology can be considered reliable, and many developments have already been achieved to obtain robust colliders, inverse Compton scattering gamma ray sources, or compact free-electron lasers (FELs). A major breakthrough could also be achieved in the future with the use of even higher accelerating structures based on W-band [117] or mm-wave linacs/THz accelerators [118], for which a gradient exceeding 200 MV/m will allow for an ultra-compact cm-scale accelerating structure with a high repetition rate, enabling fast pencil beam scanning and high dose rate delivery, thus opening up to the next generation of RT accelerators.

From the physical modelling and dose calculation point of view, Monte Carlo models and algorithms for scanned beams have already been tested and validated for VHEEs, and workflows for treatment planning with intensity-modulated scanning VHEE pencil beam therapy were demonstrated [77]. The mathematical framework to derive the dose rate at a point or voxel in a PBS field is also currently being developed for protons [119], which will most likely be applicable to scanned electron beams and serve as the basis for future algorithms with biological optimization. The optimal conformal technique is also yet to be determined. In particular, we can see that FLASH proton therapy is currently balancing between the classical conformal technique (in particular with the use of ridge filters to create depth conformation) and the shoot-through technique (which reduces many of the constraints related to the adjustment of the proton range), the latter proving capable of respecting the dose constraints to healthy tissue [120]. The possibility of easily being in FLASH conditions (e.g., a sufficiently high repetition rate while maintaining a high instantaneous dose rate) will therefore be crucial for VHEEs. Indeed, and this is the main difference with current proton machines, the instantaneous dose rate could be much higher with a linac than with a cyclotron or synchro-cyclotron, which could allow for a greater FLASH effect. However, this is a hypothesis that cannot be confirmed at the moment. If this is the case, it is very likely that VHEEs will be equally effective, and thus have a clear advantage in the implementation of FLASH radiation therapy compared to other particle beams.

## Figures and Tables

**Table 1:** Main parameters for the VHEE sources cited in this document.

Beam Parameters	CLEAR	SPARC	NLCTA
Energy (MeV)	50–220	170	50–120
Bunch charge (pC/shot)	150	60	30
Bunch length rms (ps)	0.1–10	0.87	1
Repetition rate (Hz)	0.8–10	0.1–10	0.1–10
Beam size at water phantom surface (σ mm)	1.2	3.4	2

**Table 2:** List of facilities or accelerators under development for VHEE production.

Beam Parameters	PHASER	CLARA	PITZ	Argonne	Tsinghua University
Energy (MeV)	100–200	50 (−250)	20 (−250)	6–63	45 (−350)
Bunch charge (pC/shot)	-	20–100	0.1–5000	100–10^5^	200
Bunch length rms (ps)	3.10^5^	0.3−5	30	0.3	<2
Repetition rate (Hz)	10	10 (−100)	10	0.5–10	5–50
